# Supplementary material for: High-resolution characterization of centriole distal appendage morphology and dynamics by correlative STORM and electron microscopy
Source: Nat Commun. 2019 Mar 1;10:993. doi: 10.1038/s41467-018-08216-4 (PMC6397210; doi:10.1038/s41467-018-08216-4)
Supplement: Supplementary file 1 — Supplementary Information [file 41467_2018_8216_MOESM1_ESM.pdf]

High-resolution Characterization of Centriole Distal Appendage Morphology and Dynamics by Correlative STORM and Electron Microscopy

Bowler et al.

Supplementary Information

The file contains:

1. Supplementary Figures 1-6

2. Supplementary Methods (Supplementary Figures 7-15)

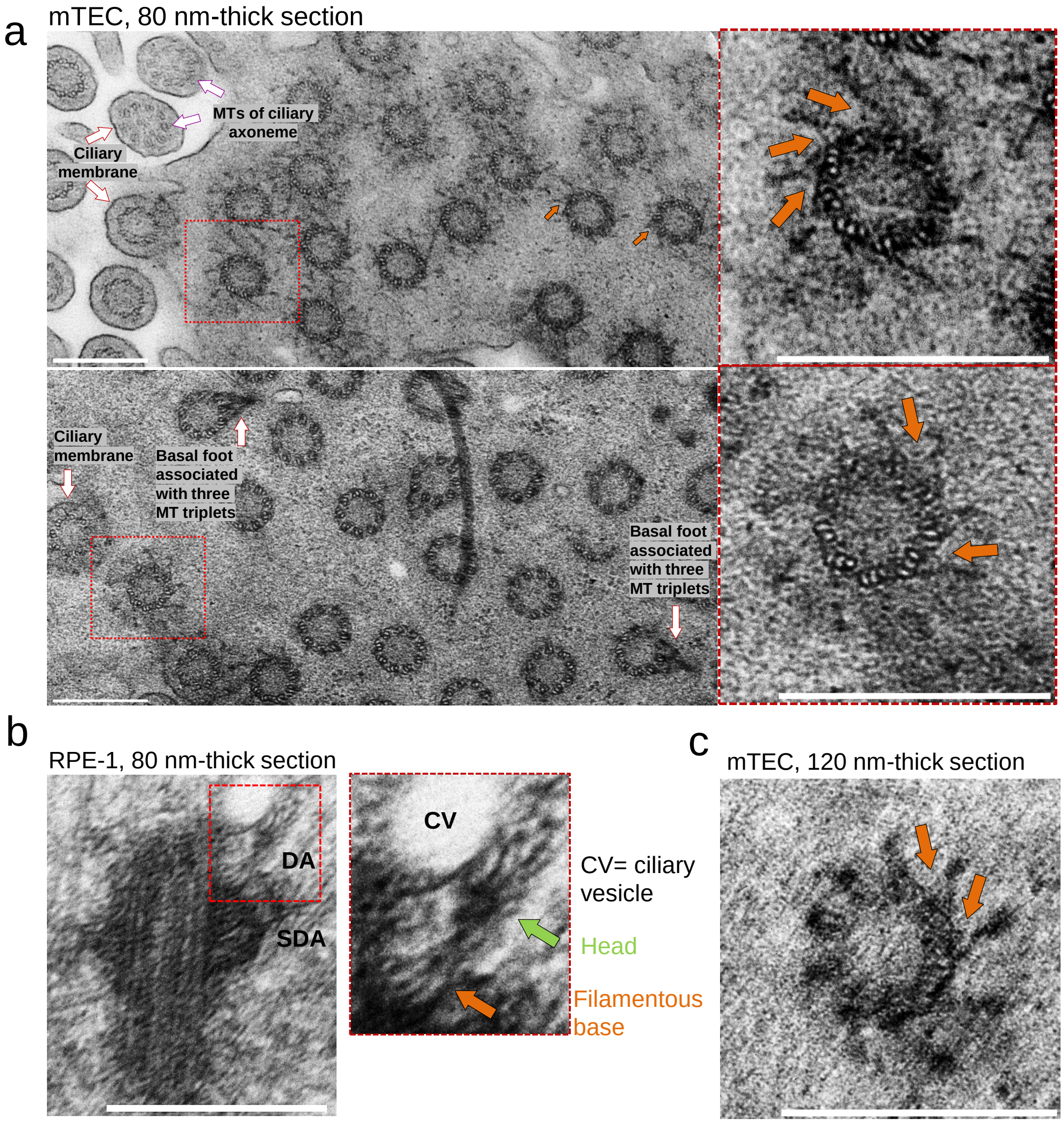

Supplementary Figure 1. Distal appendages of human and mouse associate with two centriole microtubule triplets (related to Figure 1). (**a**) Two independent 80 nm-thick sections through centrioles/basal bodies from mTEC in early stages of ciliation (ALI3), sectioned under variable angles and sectioning depths. Two basal bodies, marked by red squares are enlarged (right). Orange arrows point to the fibrous distal appendage bases which show visible association with two MT triplets. (**b**) An 80 nm-thick longitudinal section through a centriole in RPE-1 in the early stage of ciliation. A fibrous base of a distal appendage (DA, red square) is visible just above a subdistal appendage (SDA). In continuation with the fibrous

base, is an electron dense DA head, surrounded by additional material at the appendage-ciliary vesicle (CV) interface. (**c**) A 120-nm thick oblique section of a basal body from mTEC sectioned at the level of the distal appendages. Arrows point to the almost radial organization of distal appendages and their triangular fibrous bases. Scale bars: 400 nm.

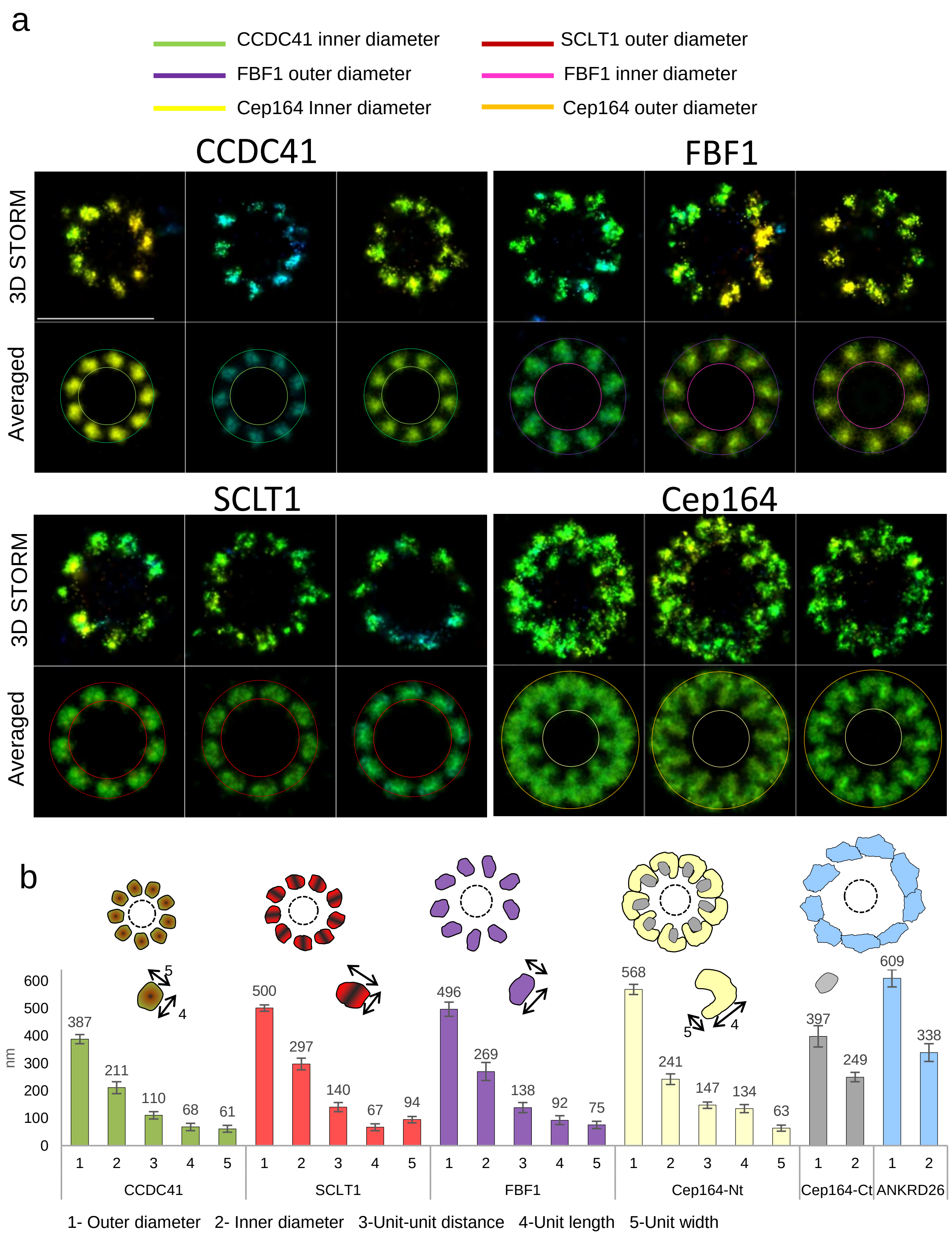

Supplementary Figure 2. Appendage proteins have a reproducible and unique nine-fold symmetrical localization pattern (related to Figure 2b). (**a**) 3D STORM signals and the averaged signals from nine appendages are shown for each protein. A mask, unique for each protein, is overlaid over averaged signals to delineate the outer and the inner diameter of the

signals, as quantified in (**b**). (**b**) Histogram plot presenting average values obtained after quantification of STORM signals associated with older mother centrioles in RPE-1 cells. Centrioles were oriented perpendicular to the coverslip. Cells were in G1 and S phase of the cell cycle. A typical size and the shape of the unit as well as its organization around the mother centriole is shown. Average±s.d. values are shown, n≥10 centrioles per protein. Scale bar: 500 nm.

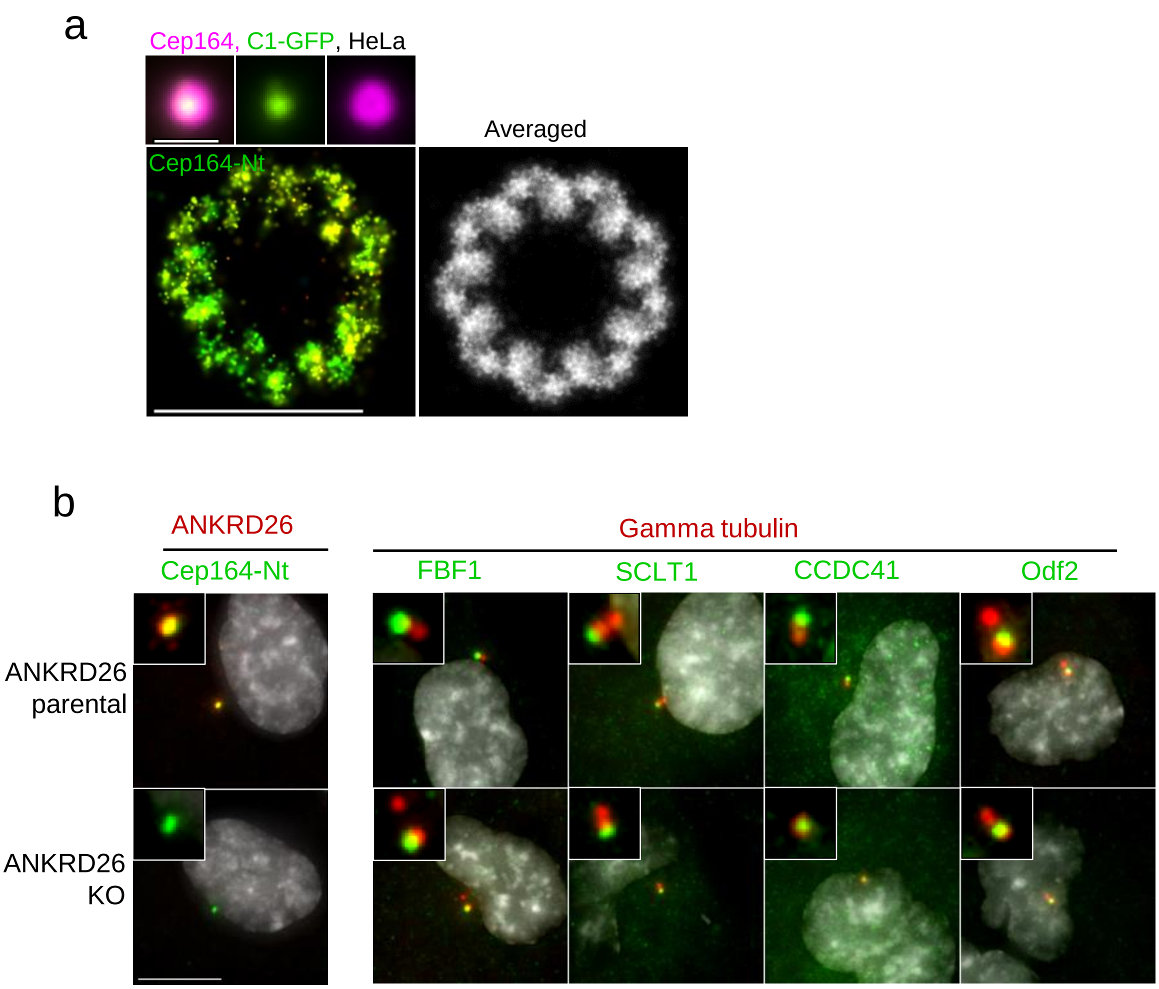

Supplementary Figure 3. 3D STORM analysis of distal appendage proteins (related to Figure 2). (**a**) STORM image of Cep164 in S phase HeLa cell. The averaged STORM signal is shown in black and white. (**b**) Immunofluorescence analysis of the DAPs in ANKRD26 KO RPE-1 cells. Left: Cep164 localizes to the centrioles in the absence of ANKRD26. Right: Cells were labeled for gamma tubulin to visualize centrosomes and for indicated DAP. Knockout of ANKRD26 does not prevent localization of other DAPs, which associate with one of the centrioles. Scale bars: wide-field images of centrioles, 1μm; wide field image in (**b**) 10 μm; STORM, 500 nm.

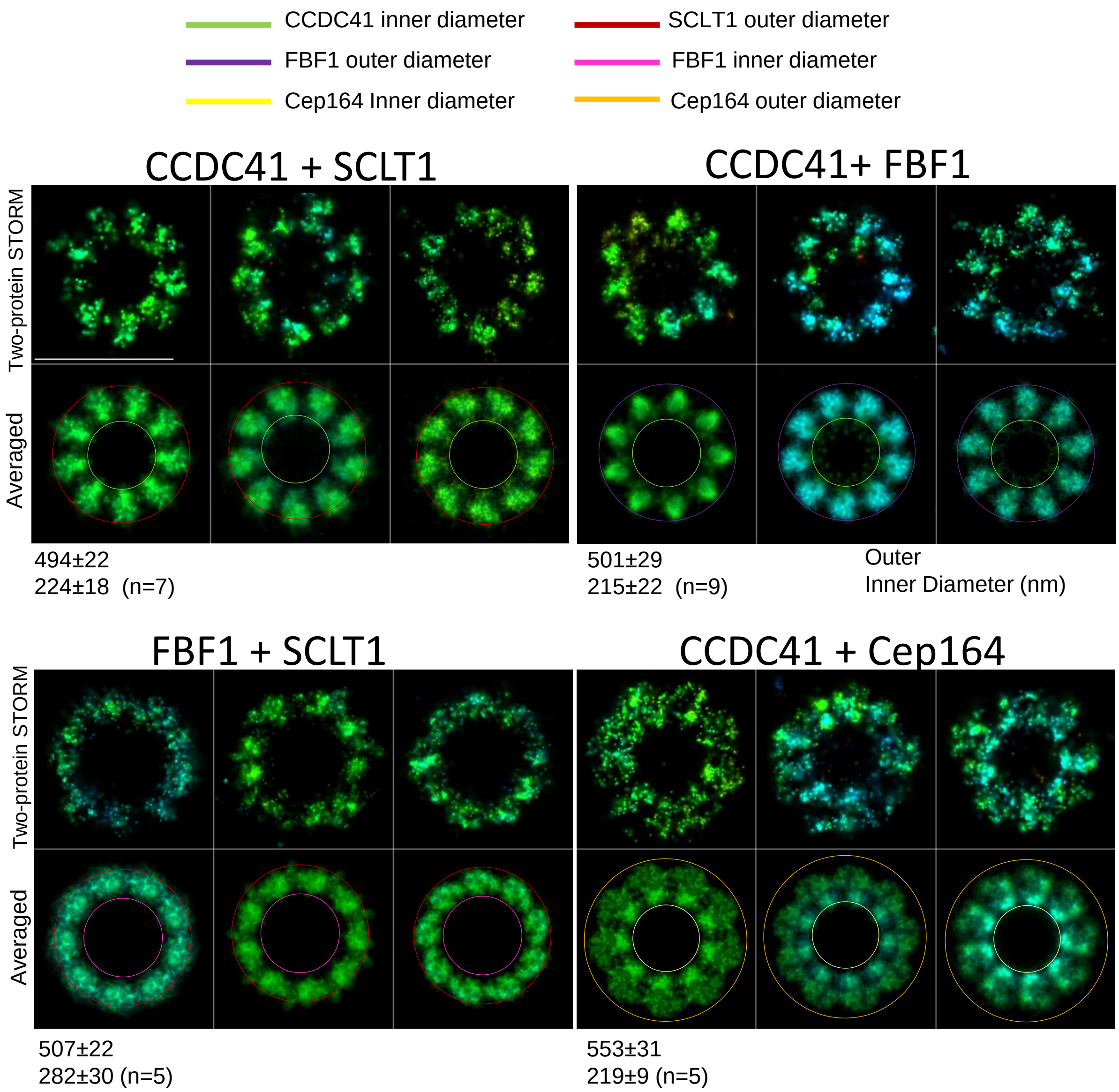

Supplementary Figure 4. A two-protein STORM analysis of distal appendage proteins (related to Figure 3c and 3d). Two protein STORM analysis of indicated DAPs. Cells were simultaneously labeled for two DAPs in one color and imaged by STORM. 3D STORM signals and the averaged signals from nine appendages are shown for each protein. A mask, is overlaid over averaged signals to delineate the outer and the inner diameter of the signals, as quantified in Supplementary Fig. 2b. Please note that a reduced concentration of Cep164 antibodies was used to reveal the position of CCDC41 signal, which is normally of a lower intensity than Cep164. The average ±s.d outer and inner diameter of the toroid is noted. Scale bar: 500 nm.

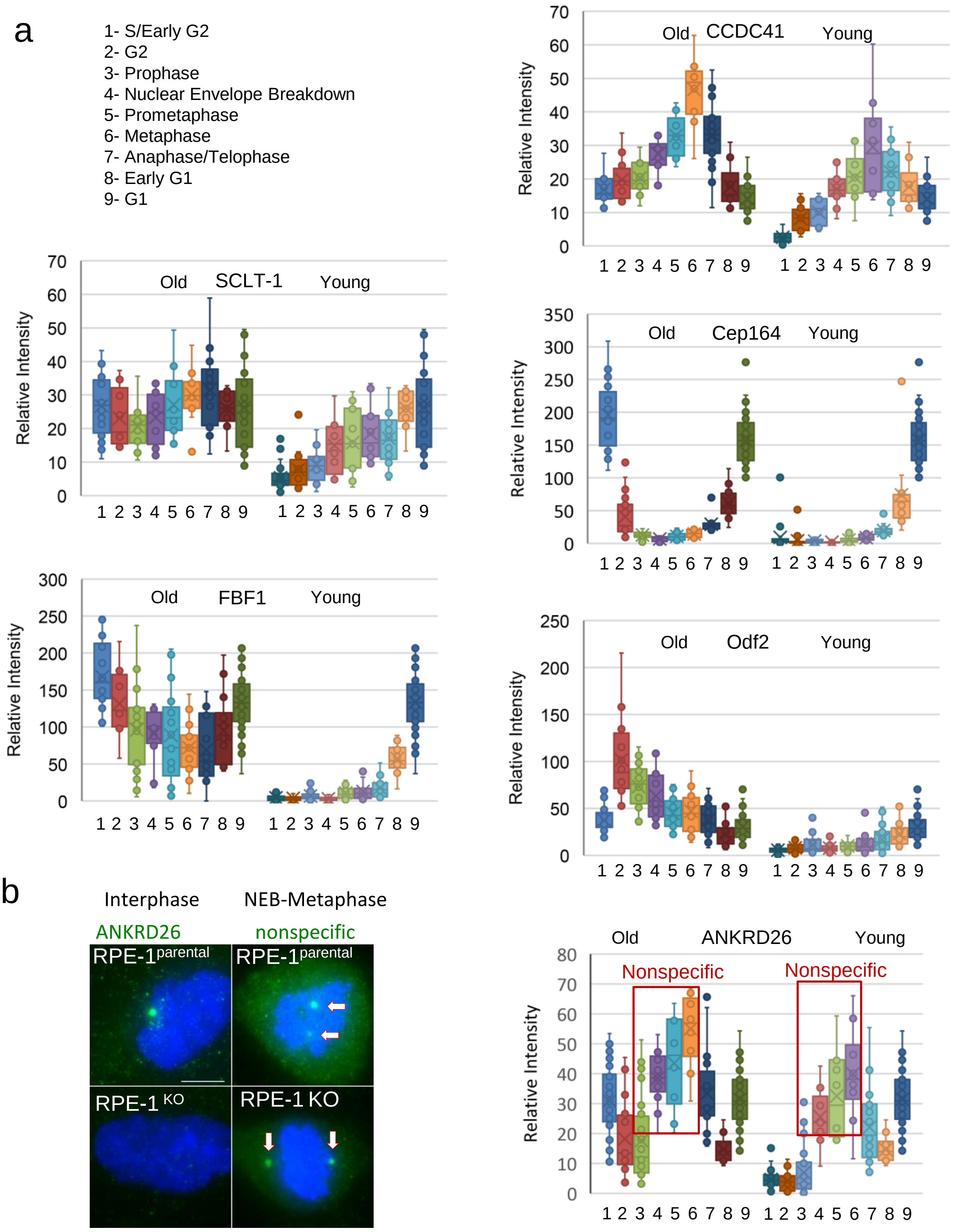

Supplementary Figure 5. The dynamics of distal appendage proteins during cell cycle (related to Figure 5 and Figure 6). (**a**) Levels of the DAPs and Odf2 associated with older mother centrioles in cycling RPE-1 cells. Box and whiskers plots represent relative intensities of indicated centriole-associated DAPs, to indicate the variation in the levels of individual DAPs.

A median line and upper and lower quartile is presented in box-and-whisker plots. n= 217 cells for Cep164, 135 cells for CCDC41, 155 for SCLT1, 176 for FBF1, 244 for Odf2. A minimum of 10 cells were measured for each point. The plots complement Figure 5a and 6a, in which only average values normalized to the G1 values are presented for clarity reasons. (**b**) Immunostaining analysis of ANKRD26 in interphase and mitotic parental and KO cells to illustrate a nonspecific staining in mitosis but not in interphase. Box and whiskers plot represents relative intensities of centriole-associated ANKRD26, to indicate cell cycle variations. However, centrosome-associated signal in mitosis is likely nonspecific. n=231 cells. A minimum of 10 cells was measured for each point. Scale bar in (**b**): 5μm. The source data underlying Supplementary Figure 5a is provided as a Source Data file.

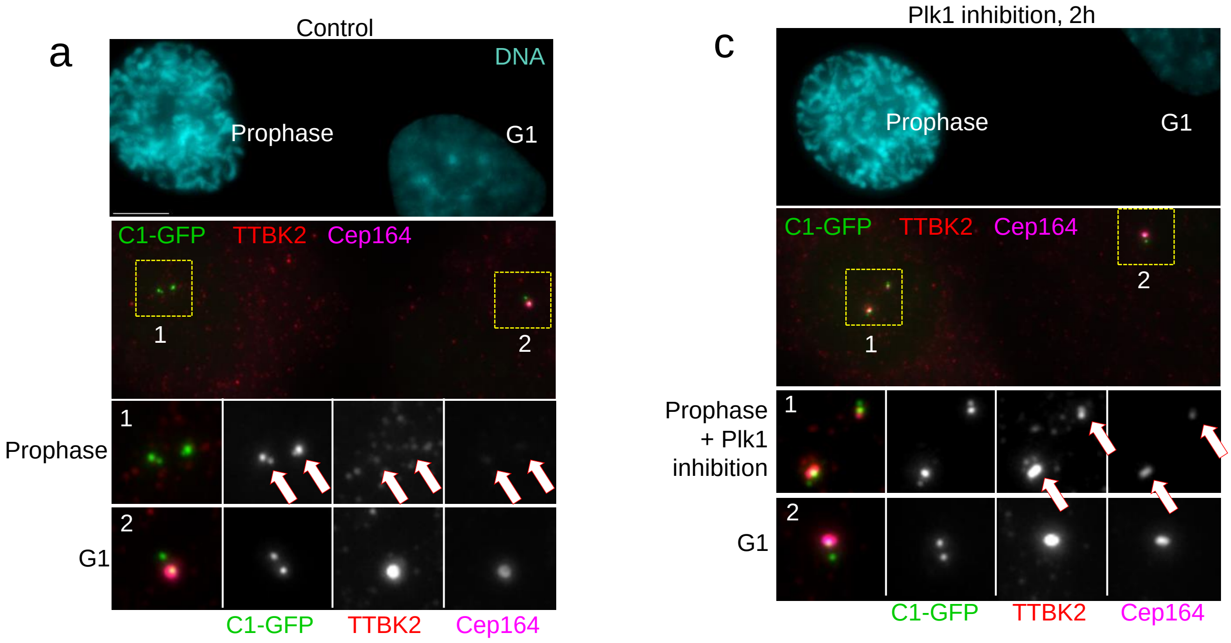

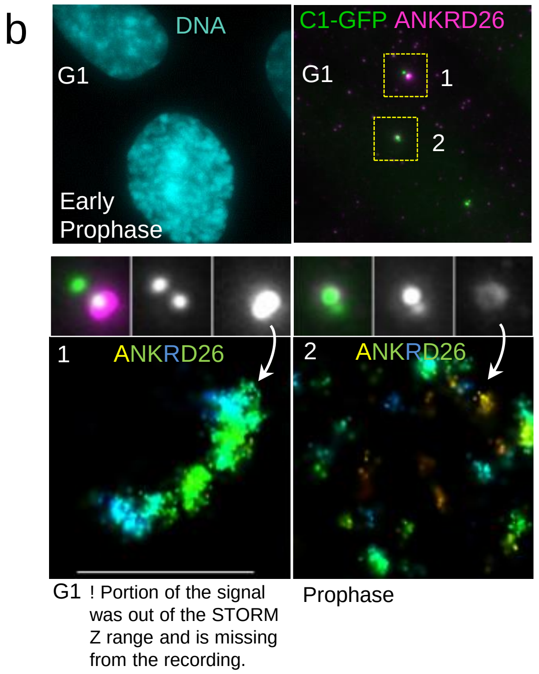

Supplementary Figure 6. Characterization of pre-mitotic dynamics of TTBK2 and ANKRD26 in RPE-1 cells (related to Figure. 7). (**a**) The levels of centriole-associated TTBK2 and Cep164 are reduced before mitosis (**b**) The levels of centrosome-associated ANKRD26 are reduced in prophase and the remaining signal appears dispersed around the centriole. (**c**) Cells were treated with Plk1 inhibitor for 2 h and immunolabeled for Cep164 and TTBK2. Both proteins are retained on the older centriole in prophase. White arrows indicate the position of the centrioles. Scale bar: 10μm for wide field images; 500 nm for STORM image in (**b**); Centrioles are enlarged images of the regions indicated in the low-magnification images.

Supplementary Methods (Supplementary Figures 7-15)

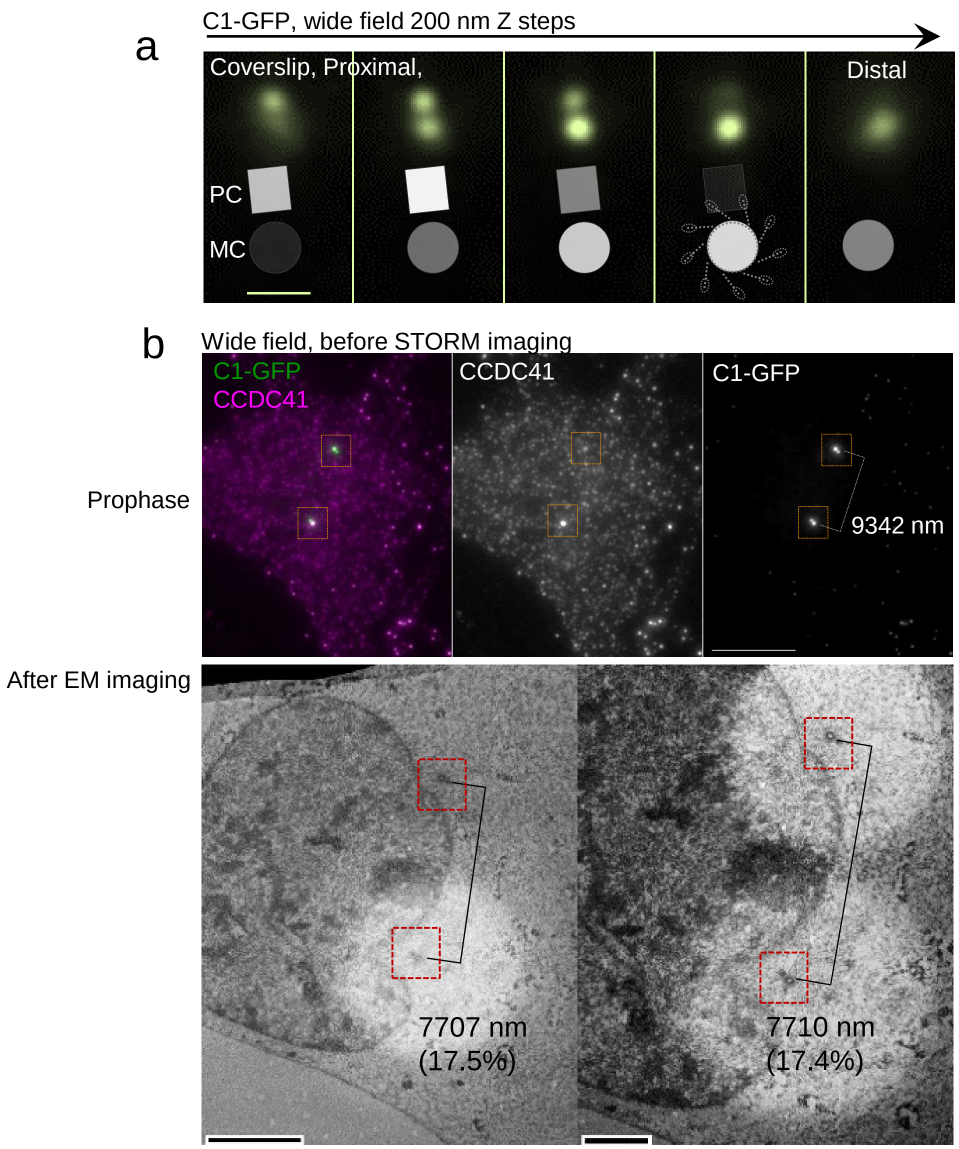

CLEM analysis of this cell is presented in Fig. 4h of the main text.

Supplementary Figure 7. Determination of centriole orientation and sample shrinkage in CLEM experiments. (**a**) The mother centriole (MC) incorporates more C1-GFP in its distal lumen than the daughter centriole (DC), which is associated with mother centriole's proximal end. Orientation of parental centrioles has been determined from a series of 200 nm-thick Z sections spanning entire centrosome, based on the appearance of the C1-GFP signals. (**b**). To determine the coefficient of sample shrinkage in CLEM experiments, the distance of the C1-GFP signals belonging to two mother centrioles were measured and compared to the distance between the centers of the centrioles on electron micrographs. The ratio of two values represents the shrinkage coefficient and was used to scale up electron micrographs in CLEM experiments before their superposition with the STORM image of the same centriole. Scale bars: 1µm (**a**), 10µm (**b** wide field), 4 and 3 µm (**b** EM).

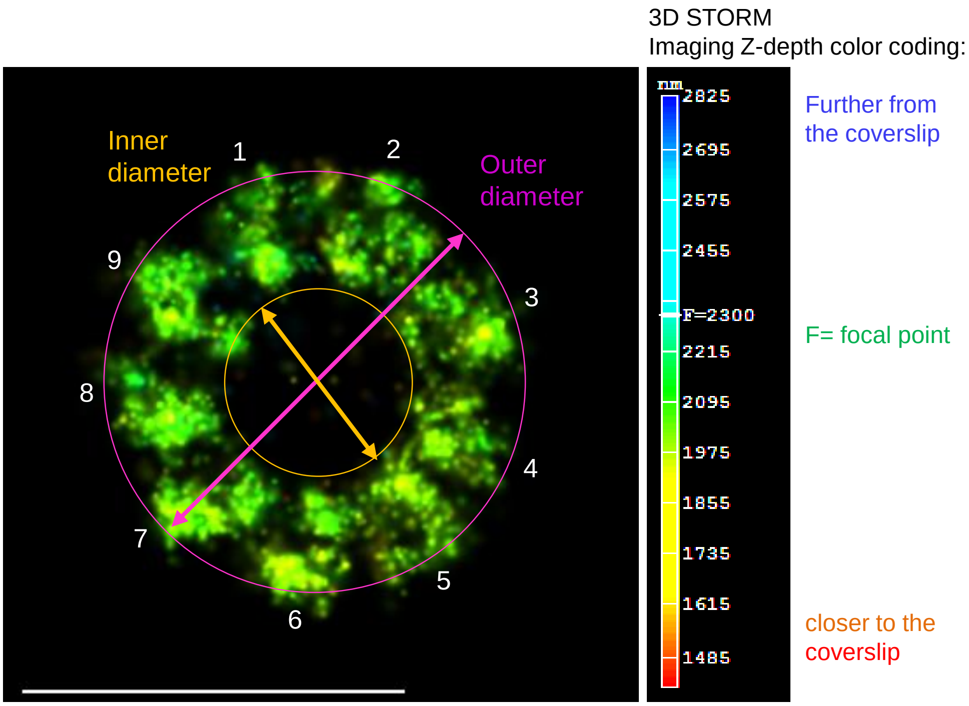

Supplementary Figure 8. A diameter of STORM signals and a Z range color-coding scheme. Scale bar: 500 nm.

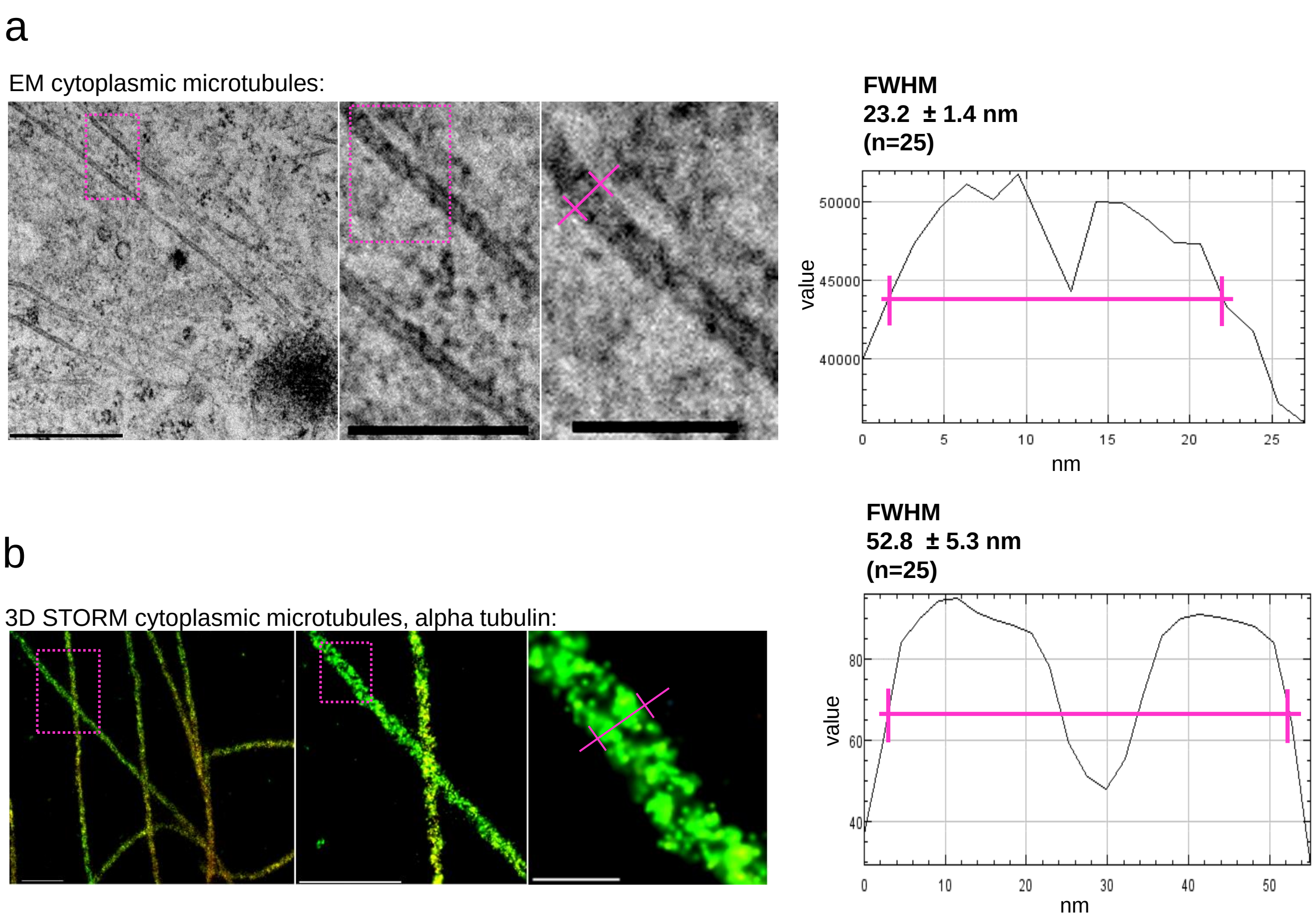

Supplementary Figure 9. Determination of resolution in 3D STORM experiments. (**a**) Cytoplasmic microtubules are polymers of alpha and beta tubulin and have relatively constant size of ~ 23 nm when analyzed by electron microscopy. (b) 3D STORM analysis of cytosolic microtubules. Cells were fixed using the fixation and immunostaining protocol used for appendage proteins, labeled by primary antibody against alpha tubulin and the secondary CF647 F(ab')2 secondary antibodies (Biotium). The average outer diameter of 3DSTORM signals measured at multiple sites of the sample was 52.8 ± 5.3 nm. This value consistent with the average diameter of the MTS (23 nm), augmented for the size of the primary and secondary FAB complexes (~15 nm on each side) From this data we determine that the resolution in our experiments is at least ~22 nm when using primary and secondary FAB antibody labeling, and <22 nm when using primary antibodies directly labeled with a fluorophore. FWHM: full width at half-maximum. Scale bars: 500, 200, 100 nm in (**a**), 500, 500 and 100 nm in (**b**).

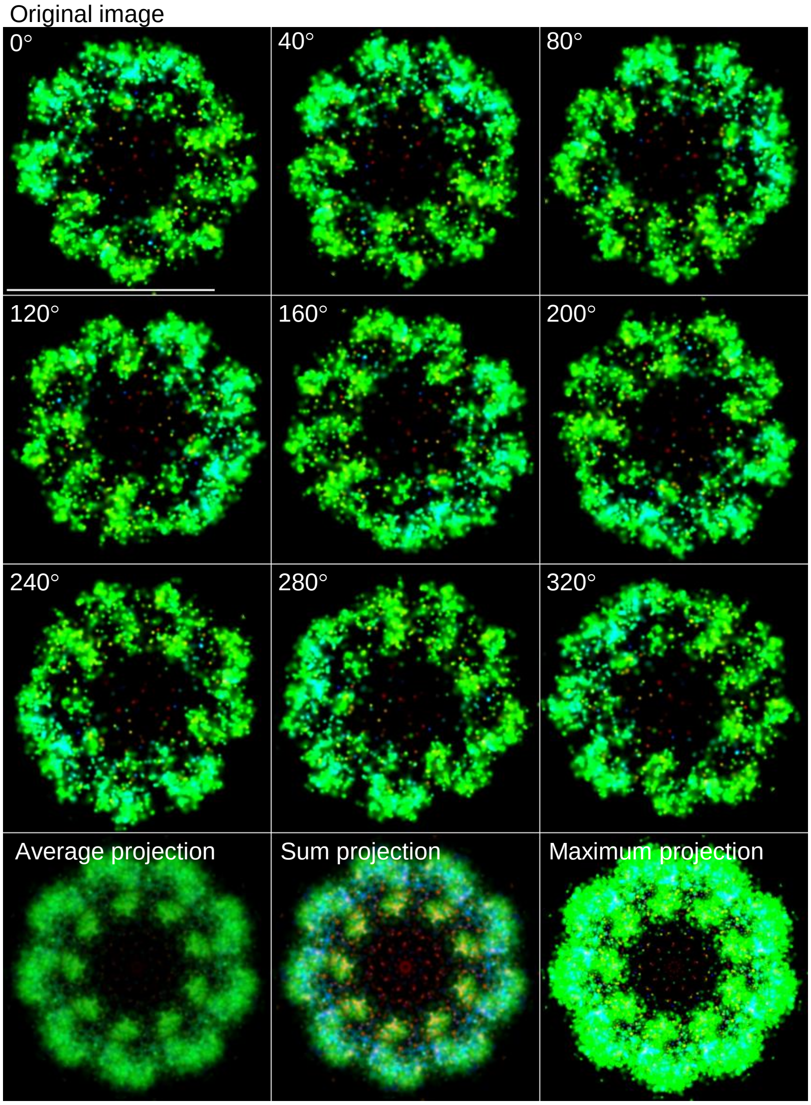

Step1:
Obtain nine rotational images

Step2:
Merge all images in one stack

Step3:
Perform averaging (by using average, sum or maximum intensity projection)

Supplementary Figure 10. Averaging Cep164 STORM signals (from Figure 2b). To average signals from 9 appendages of the same centriole, the original STORM image was rotated for 40 x n degrees (n= 0, 1, 2, 3, 4, 5, 6, 7, 8) around the physical center of the centriole. The nine images were then merged in one stack and the average, sum, and maximum projections were generated from nine orientations to emphasize the symmetry in the distribution of the protein. Scale bar: 500 nm.

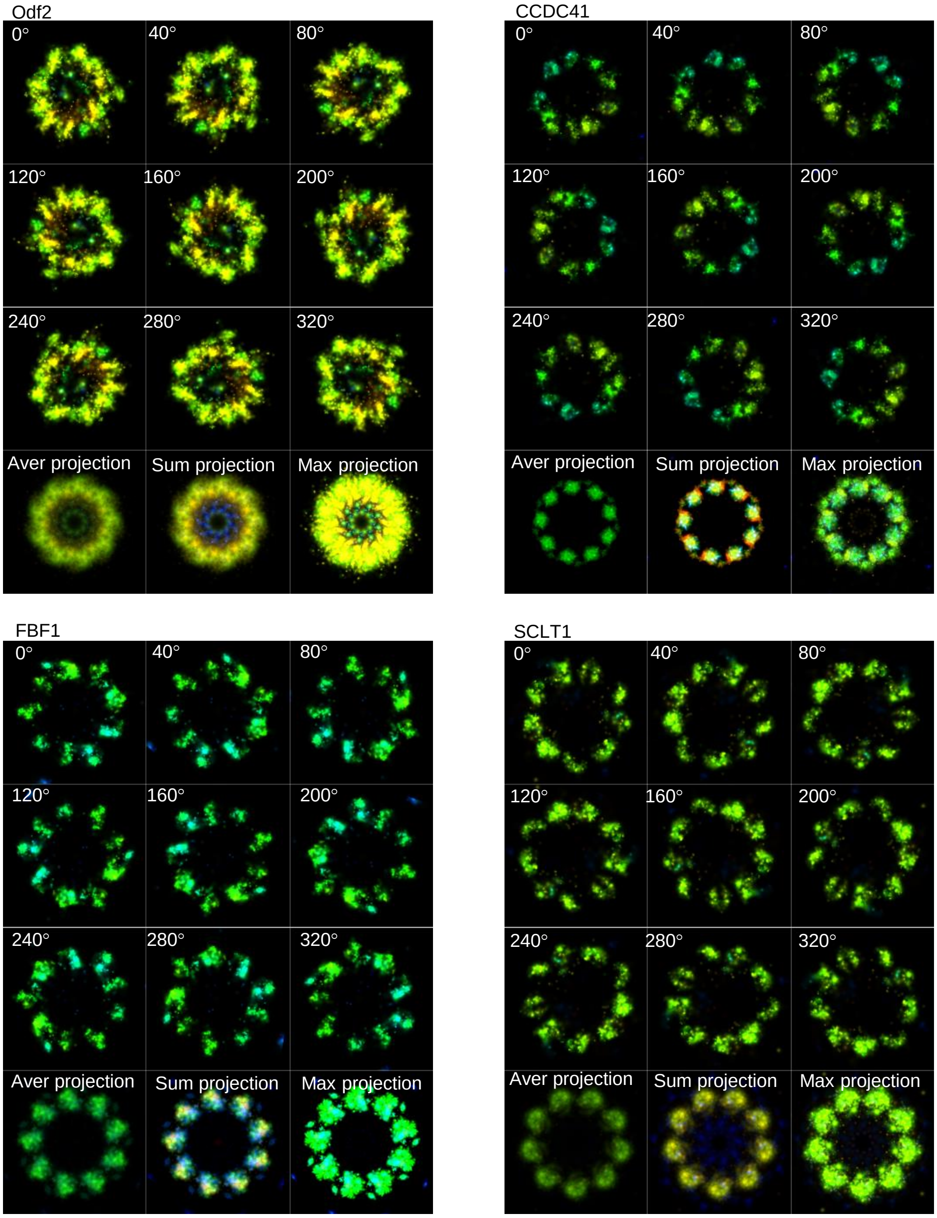

Supplementary Figure 11. Averaging STORM signals (from Figure 2b). To average signals from 9 appendages of the same centriole, the original STORM image was rotated for 40 x n degrees (n= 0, 1, 2, 3, 4, 5, 6, 7, 8) around the physical center of the centriole. The nine images were then merged in one stack and the average, sum, and maximum projections were generated.

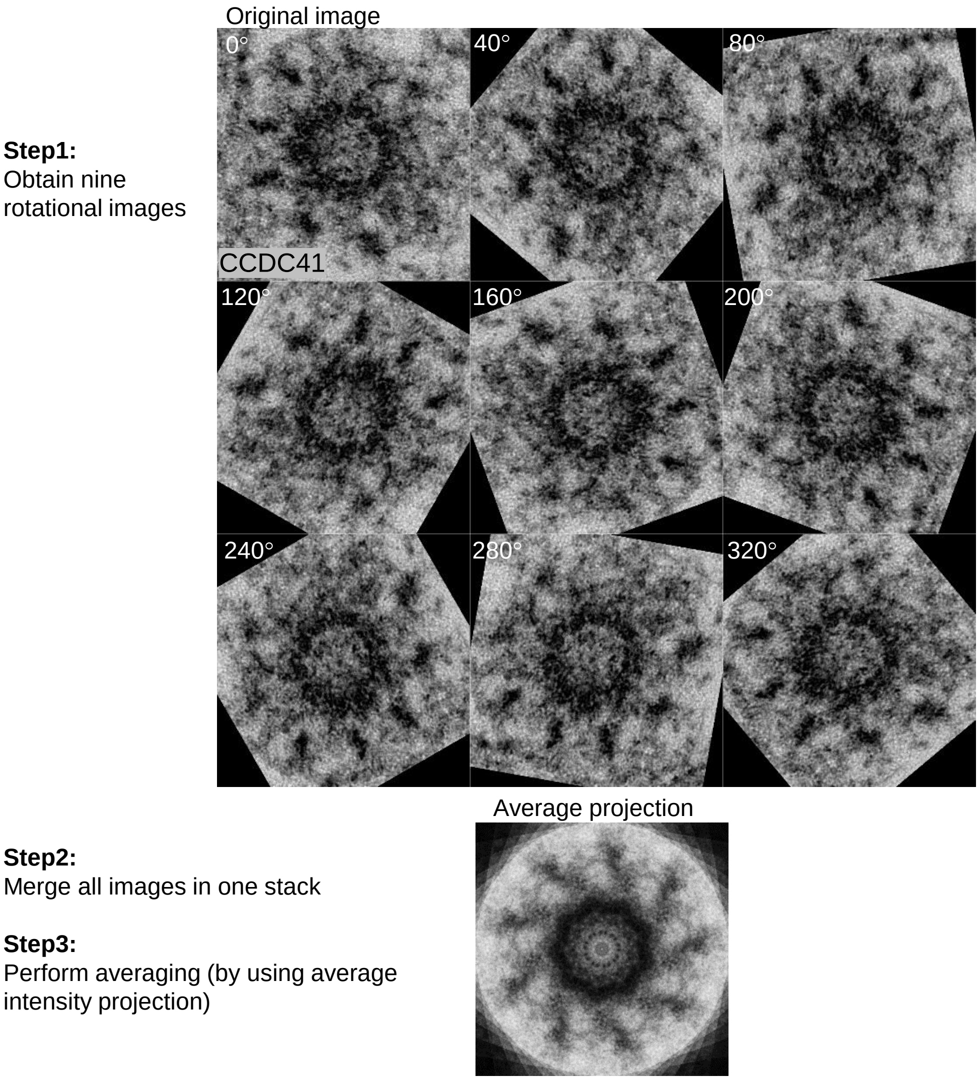

Supplementary Figure 12. Averaging of EM signals. To average signals from 9 appendages of the same centriole, the original EM micrograph was rotated for 40 x n degrees (n= 0, 1, 2, 3, 4, 5, 6, 7, 8) around the physical center of the centriole. The average projection was then generated from nine orientations.

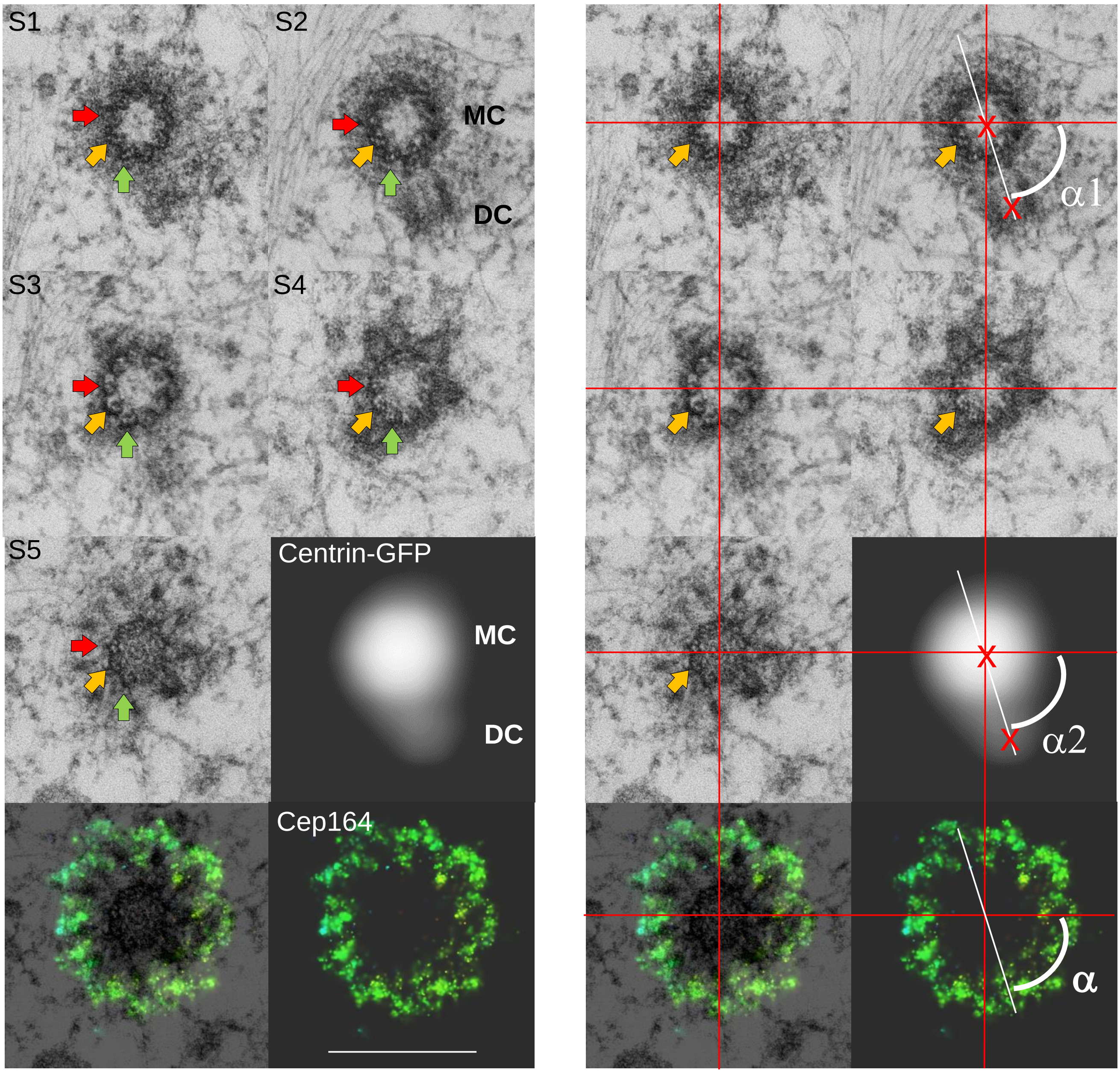

Supplementary Figure 13. Alignment of EM and 3D STORM images. The details of the alignment procedure are described in Materials and Methods. The panels illustrate aligned EM, wide-field, and STORM signal from Fig. 3a and b). Five serial sections through the centriole (S1-S5) were manually aligned. Red, yellow and green arrows point to the same microtubule set across five sections. Mother centriole (MC) is associated with the daughter centriole (DC), which is positioned orthogonally to the MC. Red cross marks are the centers of the centrioles. Red lines facilitate the appreciation of alignment. White lines are the vectors connecting the centers of two centrioles. Please note that the angles $\alpha1$ and $\alpha2$ and α are identical. Scale bar: 400 nm

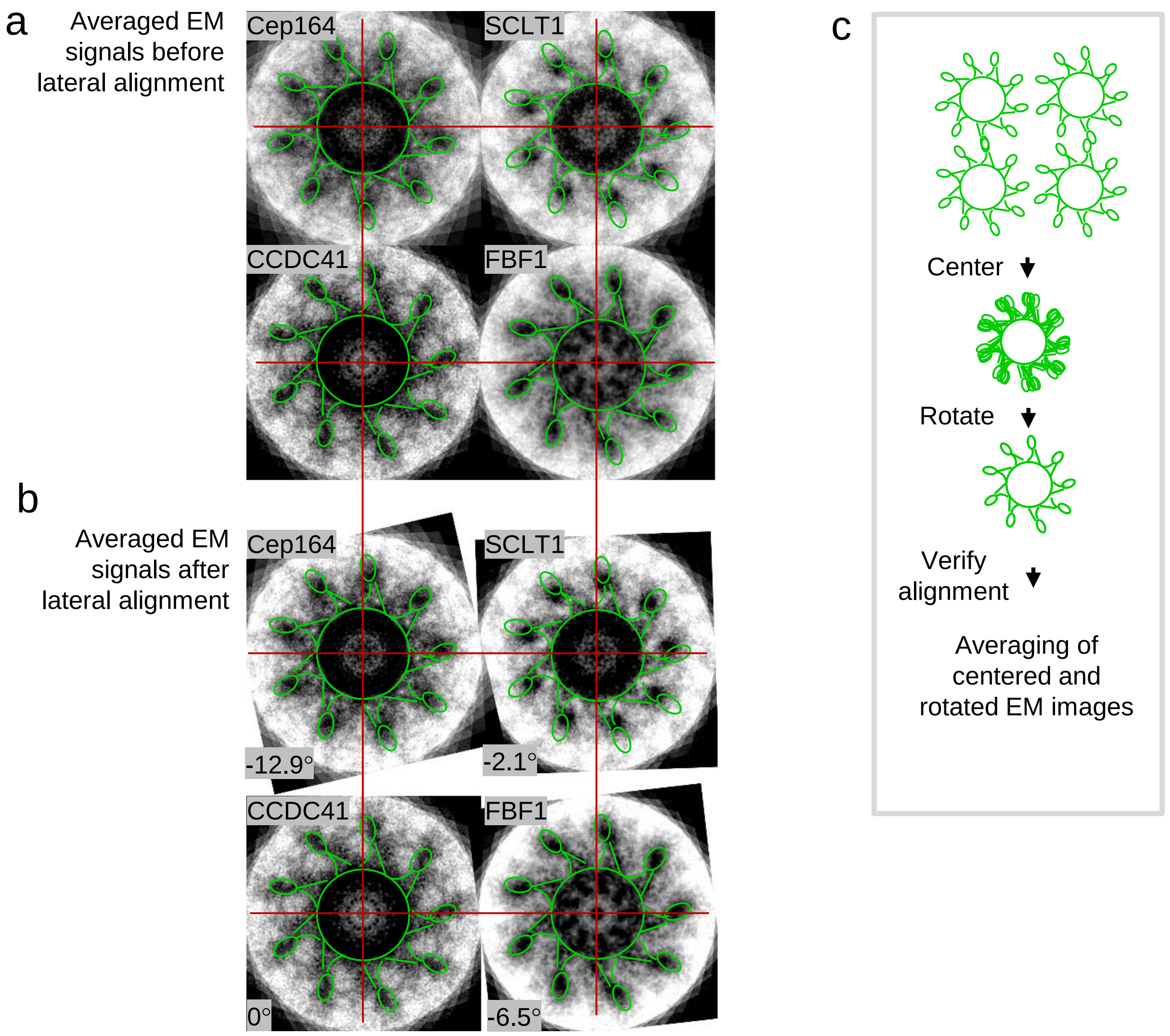

Supplementary Figure 14. Lateral alignment of averaged EM signals from CLEM experiments shown in Fig. 3a. The averaged signals of individual centrioles were generated as shown in Supplementary Methods Fig. 7. (**a**) The averaged images of four EM micrographs from Fig. 3a), before their lateral alignment. (**b**) Using Photoshop, averaged images were centered. Cep164, SCLT and FBF1 images were then rotated around the physical center of the centrioles to reach the overlap with the DA electron densities of CCDC41, which was not rotated. The averaging of aligned images was then performed to confirm the success of the alignment. The rotation angles were recorded and applied to the corresponding averaged STORM images (as illustrated in Supplementary Methods Fig. 9). The scheme (c) illustrates the procedure.

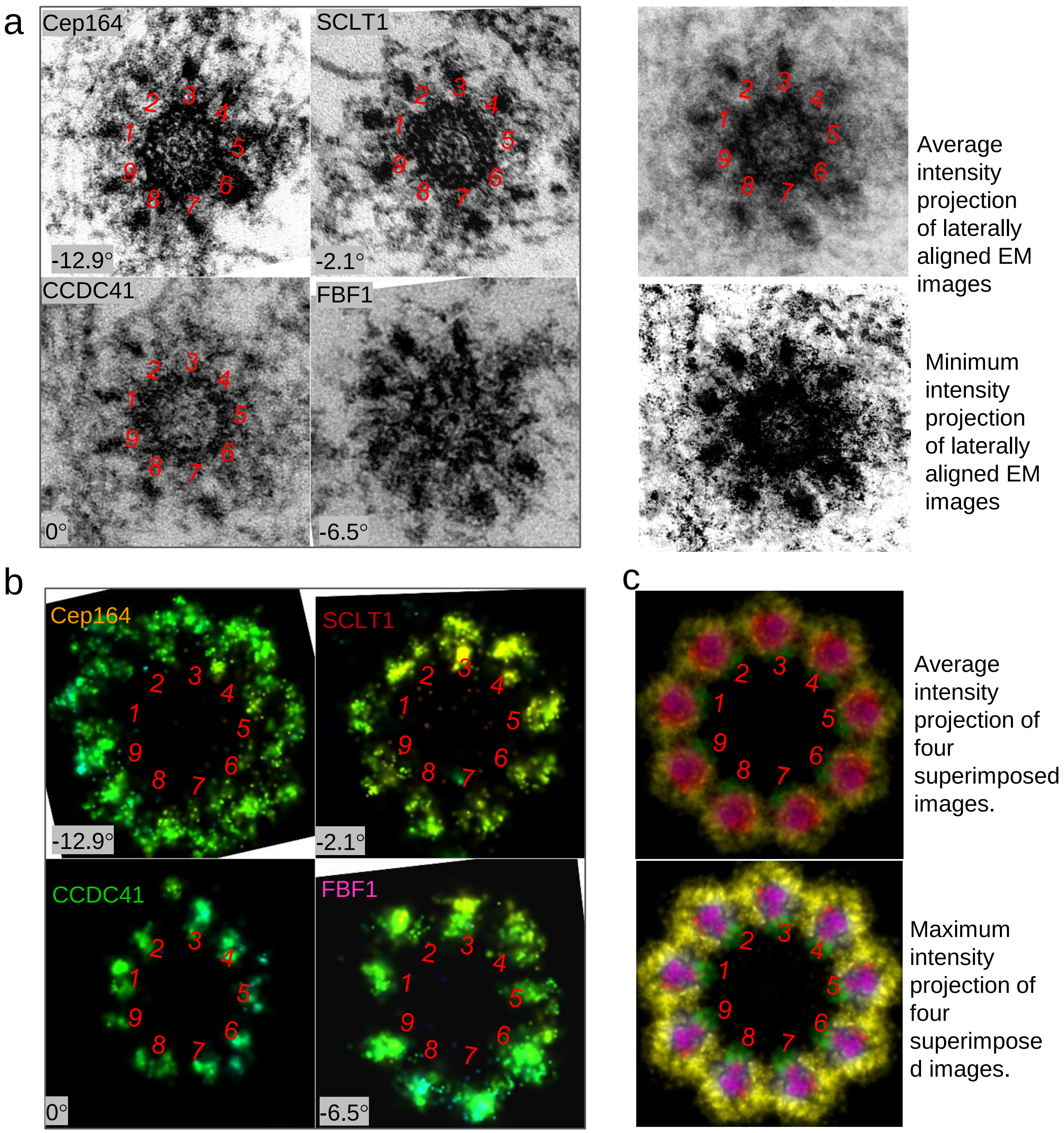

Supplementary Figure 15. Superposition of STORM signals of centrioles from CLEM experiments illustrated in Fig. 3a. (a) Lateral alignment of EM images (from Supplementary Method Fig. 8) was verified by generating average and minimum intensity projections. (**b**) STORM images were rotated using the same rotation pattern of corresponding EM images. (**c**) To superimpose STORM signals, four rotated STORM images (either original or averaged) were merged in one stack and the average and the maximum projections were generated. Red numbers are used to mark the position of individual appendages.
