# Supplementary material for: High-resolution characterization of centriole distal appendage morphology and dynamics by correlative STORM and electron microscopy
Source: Nat Commun. 2019 Mar 1;10:993. doi: 10.1038/s41467-018-08216-4 (PMC6397210; doi:10.1038/s41467-018-08216-4)
Supplement: Supplementary file 3 — Description of Additional Supplementary Files [file 41467_2018_8216_MOESM3_ESM.docx]

**Description of Supplementary Files**

**File Name**: Supplementary Movie 1

**Description:** A slice-through movie of reconstructed tomographic volume (related to Figure 1). A 120 nm-thick portion of the basal body from mTEC, containing a DA was used for electron tomography.

**File Name:** Supplementary Movie 2

**Description:** A structural model of a distal appendage (related to Figure 1). Centriole A, B, and C MTs are delineated in yellow, green and blue, respectively. A fibrous triangular DA’s base and a head density are delineated in cyan.

**File Name:** Supplementary Movie 3

**Description:** Correlative STORM/EM analysis of DAPs (related to Figure 3). The panels show a full set of 80 nm-thick serial sections, the corresponding 3D STORM image, merged STORM and EM image, and the STORM image with a scheme delineating the centriole and the DA densities.
