# Supplementary material for: High-resolution characterization of centriole distal appendage morphology and dynamics by correlative STORM and electron microscopy
Source: Nat Commun. 2019 Mar 1;10:993. doi: 10.1038/s41467-018-08216-4 (PMC6397210; doi:10.1038/s41467-018-08216-4)
Supplement: Supplementary file 7 — Reporting Summary [file 41467_2018_8216_MOESM7_ESM.pdf]

nature research

Corresponding author(s): Jadranka Loncarek

Reporting Summary

Nature Research wishes to improve the reproducibility of the work that we publish. This form provides structure for consistency and transparency in reporting. For further information on Nature Research policies, see Authors & Referees and the Editorial Policy Checklist.

Statistical parameters

When statistical analyses are reported, confirm that the following items are present in the relevant location (e.g. figure legend, table legend, main text, or Methods section).

n/a	Confirmed	
☐	☒	The exact sample size (*n*) for each experimental group/condition, given as a discrete number and unit of measurement
☐	☒	An indication of whether measurements were taken from distinct samples or whether the same sample was measured repeatedly
☐	☒	The statistical test(s) used AND whether they are one- or two-sided<br>*Only common tests should be described solely by name; describe more complex techniques in the Methods section.*
☒	☐	A description of all covariates tested
☒	☐	A description of any assumptions or corrections, such as tests of normality and adjustment for multiple comparisons
☐	☒	A full description of the statistics including central tendency (e.g. means) or other basic estimates (e.g. regression coefficient) AND variation (e.g. standard deviation) or associated estimates of uncertainty (e.g. confidence intervals)
☐	☒	For null hypothesis testing, the test statistic (e.g. *F*, *t*, *r*) with confidence intervals, effect sizes, degrees of freedom and *P* value noted<br>*Give P values as exact values whenever suitable.*
☒	☐	For Bayesian analysis, information on the choice of priors and Markov chain Monte Carlo settings
☒	☐	For hierarchical and complex designs, identification of the appropriate level for tests and full reporting of outcomes
☒	☐	Estimates of effect sizes (e.g. Cohen's *d*, Pearson's *r*), indicating how they were calculated
☐	☒	Clearly defined error bars<br>*State explicitly what error bars represent (e.g. SD, SE, CI)*

Our web collection on statistics for biologists may be useful.

Software and code

Policy information about availability of computer code

Data collection	Light microscopy data was collected in NIS elements software (Nikon, Inc.). Electron microscopy data was collected on commercial softwares integrated to transmission electron microscopes.
Data analysis	For data collection, NIS elements software was used. Images were subsequently analyzed and assembled in Fiji and Adobe Photoshop.

For manuscripts utilizing custom algorithms or software that are central to the research but not yet described in published literature, software must be made available to editors/reviewers upon request. We strongly encourage code deposition in a community repository (e.g. GitHub). See the Nature Research guidelines for submitting code & software for further information.

Data

Policy information about availability of data

All manuscripts must include a data availability statement. This statement should provide the following information, where applicable:

- Accession codes, unique identifiers, or web links for publicly available datasets
- A list of figures that have associated raw data
- A description of any restrictions on data availability

All data supporting the findings of this study are available from the corresponding author upon reasonable request.

April 2018

Field-specific reporting

Please select the best fit for your research. If you are not sure, read the appropriate sections before making your selection.

☒ Life sciences ☐ Behavioural & social sciences ☐ Ecological, evolutionary & environmental sciences

For a reference copy of the document with all sections, see nature.com/authors/policies/ReportingSummary-flat.pdf

Life sciences study design

All studies must disclose on these points even when the disclosure is negative.

Sample size	Sample size was estimated based on the variance from initial 3 or more replicates.
Data exclusions	Cells with aberrant morphology or aberrant centriole numbers (less than 5% of cell in population) were excluded from the analysis in experiments that entailed centriole imaging of quantification of centriolar proteins.
Replication	Reproducibility was obtained through initial troubleshooting and then using optimal protocols. Whenever possible, experiments were repeated by multiple researchers for cross validation of the method/approach.
Randomization	All cells with exception of cells with aberrant morphology (polyploids) or aberrant centriole number were considered for analysis. For STORM analysis, centrioles in favorable orientation were imaged. Experiments were repeated multiple times.
Blinding	Blind counting was used when quantification of cells harboring a specific phenotype (number of cilia, number of centioles associated with a specific centriole protein) was involved.

Reporting for specific materials, systems and methods

Materials & experimental systems

n/a	Involved in the study
☒	☐ Unique biological materials
☐	☒ Antibodies
☐	☒ Eukaryotic cell lines
☒	☐ Palaeontology
☒	☐ Animals and other organisms
☒	☐ Human research participants

Methods

n/a	Involved in the study
☒	☐ ChIP-seq
☒	☐ Flow cytometry
☒	☐ MRI-based neuroimaging

Antibodies

Antibodies used	Cep164 rabbit (Proteintech; 22227-1-AP, recognizing aa: 1-112) at 1:1000, FBF1 rabbit (Sigma; HPA036561, recognizing aa: 39-115) at 1:100, SCLT-1 rabbit (Sigma; HPA036561, recognizing aa:144-222) at 1:50, CCDC41/Cep83 rabbit (Sigma; HPA038161, recognizing aa: 578-677) at 1:200, ODF-2 rabbit (Proteintech; 12058-1-AP, recognizing aa: 39-181) at 1:500, Tau Tubulin Kinase 2 (Sigma; HPA018113) at 1:500, ANKRD26 (GeneTex; GTX128255) at 1:1000, acetylated tubulin mouse (Sigma; T7451) at 1:10000. CF568, CF647, or AF488 conjugated secondary antibodies (Biotium; CF647 anti-mouse 20042, CF647 anti-rabbit 20045, CF568 anti-rabbit 20099, anti-mouse 20109, Invitrogen; AF488 anti-mouse A11019, AF488 anti-rabbit A11034) were used at 1:800 dilution to label primary antibodies. In addition, Cep164 was directly conjugated with CF647 using a commercial antibody labeling kit (Biotium; 92259) and used at 1:250 dilution.
Validation	All used antibodies are commercially available and used in multiple published studies. We additionally tested the specificity of the antibodies by comparing the quality of immunolableing before and after siRNA depletion/knockdown of the target protein. Whenever possible, we compared antibodies from different vendors.

Eukaryotic cell lines

Policy information about cell lines

Cell line source(s)	RRPE-1 Centrin1-GFP, HeLaC1-GFP and mIMCD3 were from previously established stocks as detailed in Methods. mTEC primary cultures were transiently established as described in Methods.

Authentication	None of the cell lines were authenticated by our lab.
Mycoplasma contamination	Cells were regularly tested for mcoplasm contamination using microscopic methods. All cell lines were micosplasm free.
Commonly misidentified lines (See ICLAC register)	None
